# A database of geopositioned onchocerciasis prevalence data

**DOI:** 10.1038/s41597-019-0079-5

**Published:** 2019-05-22

**Authors:** Elex Hill, Jason Hall, Ian D. Letourneau, Katie Donkers, Shreya Shirude, David M. Pigott, Simon I. Hay, Elizabeth A. Cromwell

**Affiliations:** Institute for Health Metrics and Evaluation, University of Washington, 2301 5th Ave., Seattle, WA United States

**Keywords:** Parasitic infection, Epidemiology, Parasitology

## Abstract

Onchocerciasis is a neglected tropical disease with numerous symptoms and side effects, and when left untreated can lead to permanent blindness or skin disease. This database is an attempt to combine onchocerciasis prevalence data from peer-reviewed publications into a single open-source dataset. The process followed to extract and format the information has been detailed in this paper. A total of 14,043 unique location, diagnostic, age and sex-specific records from 1975–2017 have been collected, organized and marked for collapse where a single geo-position is shared between multiple records. The locations vary from single villages up to smaller administrative units and onchocerciasis control program-defined foci. This resulting database can be used to by the global health community to advance understanding of the distribution of onchocerciasis infection and disease.

## Abstract (summary)

**Table:** 

Design Type(s)	data integration objective • disease analysis objective
Measurement Type(s)	Scientific Publication
Technology Type(s)	digital curation
Factor Type(s)	geographic location • temporal_interval
Sample Characteristic(s)	Homo sapiens

Machine-accessible metadata file describing the reported data (ISA-Tab format)

## Background & Summary

Onchocerciasis is a filarial disease that can lead to permanent blindness and skin disease. Infection with *Onchocerca volvulus* is transmitted through the bite of the *Simulium* species of blackfly, which breed in fast-moving rivers. Once an individual is infected, the adult female worm circulates throughout subcutaneous connective tissues, producing thousands of larval worms (microfilariae). Microfilariae migrate into the skin and the eye, causing damage to these organs as they die, resulting in terrible itching and ocular lesions. After repeated years of exposure to microfilariae, these lesions can result in irreversible disability.

The World Health Organization estimates that approximately 200 million individuals^1^ reside in an area at risk of infection across Africa, the Americas and Yemen, with over 90% of the burden of onchocerciasis-related disease found in Africa. Large scale onchocerciasis control interventions began in 1974 with the Onchocerciasis Control Program (OCP), which employed vector control interventions throughout West Africa to reduce transmission by targeting potential breeding sites of *Simulium* black flies. In the 1980s, Mectizan (ivermectin) was demonstrated to be an effective microfilaricide, shifting the control strategy towards preventive chemotherapy via mass drug administration (MDA). In 1987, the Mectizan Donation Program began supporting national onchocerciasis control programs by supplying ivermectin free of charge. Since the inception of the donation program, MDA with ivermectin has become the primary intervention to reduce the transmission of infection. Recent evidence from the Americas^2^, Uganda^3,4^ and Sudan^5^, as well as modelling studies^6^, has shown that MDA at population coverage of at least 80% can interrupt transmission after a period of approximately 12–15 years of annual or semi-annual treatment, achieving local elimination. The success observed in these settings has led stakeholders^7,8^ to consider the elimination of onchocerciasis across Africa^9^.

The objective of this systematic review of published literature was to quantify the amount of onchocerciasis-related data available from peer-reviewed sources and aggregate those indicators into a single open source dataset. In this report, we summarize the sources identified and data extracted on onchocerciasis-related infection and disability indicators from 1975–2017, encompassing the period of implementation for control and local elimination programs among the Americas, Africa and Yemen. By presenting our results, we aim to make these data available for use in future studies of the burden on onchocerciasis-related disease as well as prevalence of infection.

## Methods

**Fig. 1:**
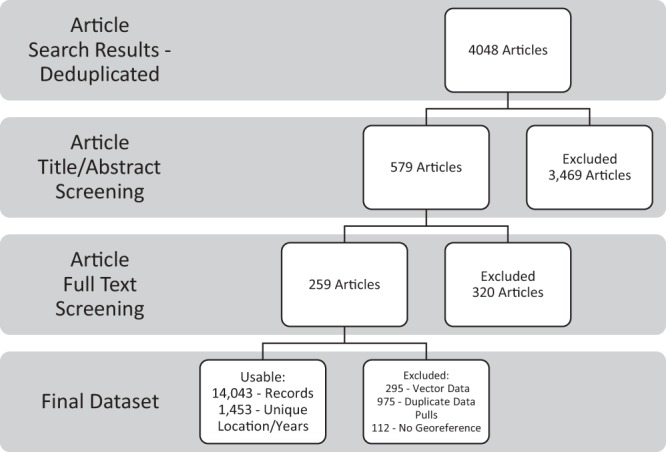
Onchocerciasis article review and data extraction flowchart. The extraction process shows the number of articles identified, screened and extracted. Each step shows how many articles or records were removed before reaching the final dataset.

The following methods outlined were designed to provide more clarity surrounding the systematic literature data collection efforts from published articles on onchocerciasis. The protocols stated here have been adapted from previously published literature extraction efforts. A guide to our extraction has been included, Fig. 1, and shows the overarching process we followed to produce this dataset.

### Data collection

Published reports of onchocerciasis were identified via searches through PubMed, Web of Science, and Scopus with the following search terms: “Oncho”, “river blindness”, “O. Volvulus”, “robles disease”, “blinding filariasis”, “coast erysipelas”, and “sowda”. The search was for all articles published about onchocerciasis prior to July 7, 2017. The exact strings used to identify articles for the systematic review can be seen in Supplementary Table 1. The search yielded 4,130 results in total, which was reduced to 2,502 after removing duplicates. The 2,502 results were then collated into a database before manually conducting title-abstract screenings. The first step of the systematic review was to implement a title/abstract screening. The purpose of this step was to remove any publications that did not report onchocerciasis prevalence among humans, case study articles, or ones solely reporting diagnostic development. A total of 579 articles underwent full text review. In order to meet the inclusion criteria, articles must have fit within the following: 1) detection of onchocerciasis in human subjects; 2) onchocerciasis cases from 1975 or later; 3) original sources only; 4) geographically representative populations only; and 5) no case-control studies. Full text review resulted in excluding 320 sources. In addition to the initial screening, all citations were reviewed to ensure relevant articles were retroactively added to the database if not already included. Through this iterative process, 18 articles that were not originally identified were retroactively added and subsequently marked for extraction. Ultimately, geographic data, as well as relevant epidemiological metadata were extracted from 259 peer-reviewed sources reporting prevalence of onchocerciasis.

### Geo-positioning of data

Location information was manually extracted at the highest resolution possible from each article using either Google Maps or ArcGIS (https://www.esri.com/en-us/home). Two classes of spatial information were evaluated: points and polygons. If location of transmission was reported to have occurred within a 5 × 5 km area, the geography was defined as a point, and represented by a specific latitude and longitude. This definition of a point referencing an area smaller than 5 × 5 km was done to be compatible with satellite imagery, typically resolved at 5 km × 5 km for global analyses.

If location of transmission occurred within an area greater than 5 × 5 km (e.g. a large city), or if the location of transmission was less clear but known to have occurred in a general area (e.g. a province), a polygon was assigned to cover the region of the reported occurrence. In instances where the author’s spelling of a location differed from ArcGIS or Google Maps, contextual information was utilized in order to determine the location. Where authors provided maps, these were digitized using ArcGIS.

Three different types of polygons were used: known administrative boundaries, buffers, and custom polygons. For governorates, districts, or regions, the relevant administrative unit (sourced from the Global Administrative Unit Layers curated by the Food and Agricultural Organization of the UN^10^) was paired with the record. For cities and regions without corresponding administrative units, a buffer was created to encompass the area. Buffers were created by generating a circle that encompassed the entirety of the region of interest, using Google Maps to evaluate the required radius. Custom polygons were created in ArcGIS for areas with unspecified boundaries, such as various groups of communities and specific rivers that had been surveyed along. For subsequent re-identification, each polygon is assigned a unique code within a defined shapefile representing the spatial extent.

## Data Records

The database has been made publicly available: Open Science Framework^11^ (OSF). Each row represents a unique location, year, diagnostic, age, and sex combination of data. A summary table of the number of records by diagnostic and location are presented in Table 1. The database contains the following fields:

1. NID: Unique source identifier number that connects to the survey or paper that can be found through the Institute for Health Metrics and Evaluation’s (IHME) Global Health Data Exchange (http://ghdx.healthdata.org/).
2. SITE_MEMO: A character string that details out the breakdown of the record’s location.
3. LOC_GROUP: A unique identifying number that groups record rows that are georeferenced to the same location.
4. LOC_UNIQ: A unique identifying number that groups record rows within a unique LOC_GROUP number that share the SITE_MEMO. This is done for collapse purposes when a location could not be individually georeferenced.
5. LOC_SPEC: A character string to identify within each unique LOC_GROUP and LOC_UNIQ combo to signify whether that row represents a portion of the location total by a Sex, Age, or Sex Age subset.
6. COUNTRY: A unique character string used by IHME to identify country and or subnational location within a country.
7. POINT: Whether the record has been georeferenced to a point or a polygon. 1 = point; 0 = polygon.
8. LAT: If POINT is 1, this is a decimal point value to represent the latitude of the point, otherwise this value will be NA.
9. LONG: If POINT is 1, this is a decimal point value to represent the longitude of the point, otherwise this value will be NA.
10. POLY_REFERENCE: If point is 0, this is a character string to represent the name of the shapefile that contains the georeferenced shape for this record.
11. POLY_ID_FIELD_NAME: If POINT is 0, this is a character string to represent the name of the column in the shapefile that contains the unique identifier to connect the record to the polygon within the specified shapefile in POLY_REFERENCE.
12. POLY_ID: If POINT is 0, this is a unique identifying numeric number to reference within the shapefile and column specified in POLY_REFERENCE and POLY_ID_FIELD_NAME to the polygon this record has been georeferenced to.
13. AGE_START: A number that represents the start of the age range tested for this record. If no age is provided, assumed value of 0.
14. AGE_END: A number that represents the end of the age range tested for this record. If no age is provided, assumed value of 99.
15. SEX: A character string to represent which sex was tested in this record. Possible Values: Both, Female, Male.
16. YEAR_START: Starting year of the data collected from the study. If no year provided, assumed start year to be 3 years before the publication date.
17. MONTH_START: If provided, a numeric value between 1 and 12 to represent the starting month, otherwise it is given the value NA.
18. YEAR_END: Ending year of the data collected from the study. If no year provided, assumed end year to be 1 year before the publication date.
19. MONTH_END: If provided, a numeric value between 1 and 12 to represent the ending month, otherwise it is given the value NA.
20. DX_CODE: A numeric value (integer) to represent the test for the presence or symptoms of onchocerciasis performed in this record. A table describing specific diagnostic codes has been included in the OSF data upload and in Supplementary Table 2.
21. DX_GROUP: A character string that represents what diagnostic grouping this record belongs to. In the table of specific diagnostic names to codes you can find how codes were grouped. Possible values: Prevalence - ss (Skin Snip), nod (Nodules), sero (Serology), otherPrev (Other Prevalence); Sequelae - skin (Skin Symptoms), eye (Eye Symptoms).
22. N: A numeric value representing the number of surveyed participants for this record.
23. CASES: A whole numeric value representing the number of persons who tested positive for this record.

**Table 1:** Summary of counts of onchocerciasis diagnostic records by region and country.

Location	Prevalence Records				Sequelae Records		Total Records
	Skin Snip	Nodules	Serology	Other	Skin Symptoms	Eye Symptoms	
**Africa**	**2**,**474**	**1**,**605**	**195**	**199**	**5**,**164**	**2**,**015**	**11**,**652**
Angola	0	30	0	0	0	0	30
Benin	64	36	0	0	0	4	104
Burkina Faso	60	9	0	0	0	64	133
Burundi	3	15	0	110	21	5	154
Cameroon	328	863	21	66	3,621	681	5,580
Central African Republic	26	9	0	1	5	28	69
Congo	5	31	0	0	31	0	67
Cote d’Ivoire	47	1	4	0	0	28	80
Democratic Republic of the Congo	6	2	0	18	6	19	51
Equatorial Guinea	69	16	3	0	39	23	150
Ethiopia	201	19	0	0	81	9	310
Gabon	0	8	0	1	36	11	56
Ghana	29	22	0	0	4	49	104
Guinea	19	0	0	0	0	0	19
Liberia	224	12	0	0	0	22	258
Malawi	2	0	0	0	0	1	3
Mali	56	0	4	2	0	0	62
Nigeria	799	329	23	0	985	544	2,680
Senegal	52	0	14	1	0	0	67
Sierra Leone	210	82	0	0	161	158	611
South Sudan	1	1	0	0	8	1	11
Sudan	4	11	4	0	41	6	66
Tanzania	83	18	0	0	85	193	379
Togo	95	2	68	0	0	17	182
Uganda	91	89	54	0	40	152	426
**Latin America**	**732**	**248**	**243**	**41**	**224**	**903**	**2**,**391**
Brazil	38	0	13	0	0	3	54
Colombia	4	0	0	0	0	0	4
Ecuador	198	17	69	1	22	11	318
Guatemala	319	190	121	21	187	845	1,683
Mexico	15	0	31	0	0	10	56
Venezuela	158	41	9	19	15	34	276
**TOTAL**	**3**,**206**	**1**,**853**	**438**	**240**	**5**,**388**	**2**,**918**	**14**,**043**

Summary table of individual record counts found in each country sorted by referenced diagnostic type.

We have reviewed, standardized, and grouped all the diagnostics we extracted in this process and have detailed out the translation between diagnostic code and diagnostic name or diagnostic group in three tables included in the Global Health Data Exchange. In total, we extracted information on 120 diagnostics; 21 prevalence tests, 41 skin symptoms, and 58 eye symptoms. The conversion tables can be found in the OSF data upload and in Supplementary Table 2.

## Technical Validation

The data validation was managed by a senior extractor who supervised the data entry performed by six data extraction analysts. Potential duplicate data records were investigated and removed if necessary, formatting and naming conventions were standardized across the data, and a new numbering system for collapse purposes was created programmatically due to miscommunication during the initial extraction. We also standardized and removed duplicate diagnostic test categories in the final dataset.

**Fig. 2:**
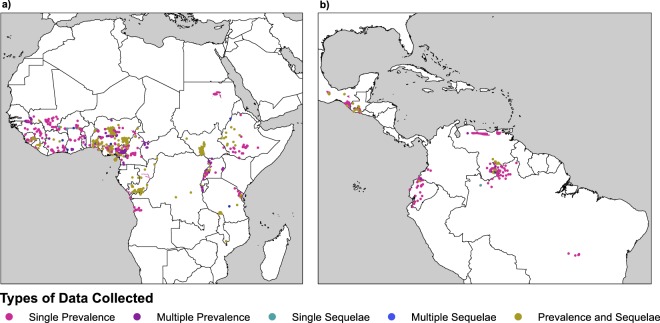
Locations of onchocerciasis data from 1975–2017 by diagnostics groupings. (**a**) Data in Africa (**b**) data in Latin America. Maps that show each individual point or polygon colored by the type and amount of diagnostics reported in that single location. Prevalence data includes skin snips, nodule, and serology tests. Sequelae data captures any onchocerciasis-related morbidity for skin or eye problems, such as vision impairment or onchodermatitis symptoms. (**a**) Dataset records that have been georeferenced inside of Africa. (**b**) Dataset records that have been georeferenced inside of the Americas.

The georeferencing was verified by putting all the points and polygons onto a map and checking by year to see if there was any overlap between the locations. If overlap was found, the georeferencing would be manually double checked to ensure accuracy and remove redundancy. Every single polygon location was verified as well to ensure we had the smallest, most accurate polygon possible to represent the area surveyed. A finalized map of all points and polygons collected has been broken down into two maps, one of Africa (Fig. 2a) and one of the Americas (Fig. 2b). The geo-locations have been color coded to represent which locations have a specific type or types of diagnostics collected in that area.

The final georeferenced dataset is 14,043 records. The 112 records that we were unable to georeferenced to a subnational location have been kept and georeferenced to the country from which they were collected. A summary table of what countries these records belong to can be seen in Table 2.

**Table 2:** Summary of counts of non-georeferenced onchocerciasis data records by county.

Country	Count
Burundi	4
Central African Republic	3
Cameroon	10
Democratic Republic of Congo	3
Congo	2
Ethiopia	4
Guatemala	16
Liberia	1
Mali	2
Malawi	4
Nigera	21
Chad	12
Tanzania	10
Uganda	20

Summary table of individual record counts found in each country that could not be georeferenced. We were unable to reasonably or accurately locate a total of 112 records.

## Usage Notes

We provide a comprehensive dataset with onchocerciasis infection prevalence and related skin and eye disease. As national programs consider expanding mass drug administration of Ivermectin to achieve the elimination of onchocerciasis infection, national onchocerciasis elimination committees^12^ are tasked with compiling both current and historical data. The publication of this systematic review will enable stakeholders to review the published literature for locations, years or indicators of interest. Users should note that age-specific data are stored as reported in the original source, not aggregated by location or year.

For grouping purposes, we have included three variables –LOC_GROUP, LOC_UNIQ, and LOC_SPEC. These should be used by first pulling the diagnostic group information from the data the user is interested in and then collapsing by LOC_SPEC into LOC_UNIQ into LOC_GROUP.

This systematic review was conducted for the purposes of modeling the burden of onchocerciasis for the Global Burden of Disease Study (GBD). Since the GBD is implemented on an annual basis, the dataset will be updated on a routine basis to account for newly published data.

## Supplementary Information

### ISA-Tab metadata file

Download metadata file

### Supplementary information

Supplementary Information
