# Supplementary material for: A database of geopositioned onchocerciasis prevalence data
Source: Sci Data. 2019 May 22;6:67. doi: 10.1038/s41597-019-0079-5 (PMC6531454; doi:10.1038/s41597-019-0079-5)
Supplement: Supplementary file 2 — Supplementary Information [file 41597_2019_79_MOESM2_ESM.docx]

Supplementary Index for *A database of geopositioned onchocerciasis prevalence data*

Table of Contents

Supplementary Table 1: Search strings used on specific article database websites **2**

Supplementary Table 2: Comprehensive list of all diagnostics and corresponding codes **4**

**Supplementary Table 1: Search strings used on specific article database websites**

| **Site** | **Search String** |
| --- | --- |
| [Pubmed](https://www.ncbi.nlm.nih.gov/pubmed/) (https://www.ncbi.nlm.nih.gov/  pubmed/) | (oncho*[Title/Abstract] OR "river blindness"[Title/Abstract] OR "O. volvulus"[Title/Abstract] OR "robles disease"[Title/Abstract] OR "blinding filariasis"[Title/Abstract] OR "coast erysipelas"[Title/Abstract] OR “sowda” [Title/Abstract] OR “nodding syndrome”[Title/Abstract]) AND (“1975”[Date – Publication] : “2016”[Date – Publication]) AND (epidemiology[Title/Abstract] OR prevalence[Title/Abstract] OR incidence[Title/Abstract] OR surveillance[Title/Abstract] OR”MDA”[Title/Abstract] OR “Mass Drug Administration”[Title/Abstract] OR “Community-directed treatment with ivermectin”[Title/Abstract] OR “CDTI”[Title/Abstract] OR “mass treatment”[Title/Abstract] OR “multiple ivermectin treatments”[Title/Abstract] OR “monthly doses of ivermectin”[Title/Abstract] OR “large scale treatment”[Title/Abstract] OR REMO[Title/Abstract] OR “Rapid epidemiological mapping of onchocerciasis”[Title/Abstract] OR APOC[Title/Abstract] OR “African Programme for Onchocerciasis Control”[Title/Abstract] OR OCP[Title/Abstract] OR “Onchocerciasis Control Programme”[Title/Abstract]) NOT(Animals[MeSH] NOT Humans[MeSH]) |
| [Web of Science](https://clarivate.com/products/web-of-science/) (https://clarivate.com/products/  web-of-science/) | TS=(oncho* OR "river blindness" OR "O. volvulus" OR "robles disease" OR "blinding filariasis" OR "coast erysipelas" OR sowda OR “nodding syndrome”) AND TS=(epidemiology OR prevalence  OR incidence  OR surveillance OR MDA OR “Mass Drug Administration” OR “Community-directed treatment with ivermectin” OR CDTI OR “mass treatment” OR “multiple ivermectin treatments” OR “monthly doses of ivermectin” OR “large scale treatment” OR REMO OR “Rapid epidemiological mapping of onchocerciasis” OR APOC OR “African Programme for Onchocerciasis Control” OR OCP OR “Onchocerciasis Control Programme”) NOT TS=((Animals NOT Humans)) |
| [Scopus](https://www.scopus.com/search/form.uri?display=basic)  (https://www.scopus.com) | (TITLE-ABS-KEY(oncho* OR "river blindness" OR "O. volvulus" OR "robles disease" OR "blinding filariasis" OR "coast erysipelas")) AND TITLE-ABS-KEY(epidemiology OR prevalence OR incidence OR surveillance OR MDA OR "Mass Drug Administration" OR "Community-directed treatment with ivermectin" OR CDTI OR "mass treatment" OR "multiple ivermectin treatments" OR "monthly doses of ivermectin" OR "large scale treatment" OR REMO OR "Rapid epidemiological mapping of onchocerciasis" OR APOC OR "African Programme for Onchocerciasis Control" OR OCP OR "Onchocerciasis Control Programme") AND NOT KEY(Animals NOT Humans) AND PUBYEAR > 1975 |

Caption: A collection of all search strings and corresponding article database websites that were used to start this extraction process.

**Supplementary Table 2: Comprehensive list of all diagnostics and corresponding codes**

| **Diagnostic Group** | **Diagnostic Code** | **Diagnostic Name** |
| --- | --- | --- |
| ss | 1 | Skin snip + microscopy |
| ss | 2 | Skin snip + PCR |
| ss | 9 | Skin snip, no further details |
| ss | 10 | Skin Snip - Membrane Filter Concentration Technique |
| nod | 30 | Any palpable nodules |
| nod | 92 | Skin Snip w/PCR + Any Palpale Nodules |
| nod | 95 | Skin Snip (non-PCR) + Any Palpale Nodules |
| nod | 39 | Nodules, no further info |
| sero | 100 | ELISA OV16 |
| sero | 102 | Ov16 RDT |
| sero | 416 | ELISA OvMBP16, OvMBP11, OvMBP7 |
| sero | 426 | ELISA Ov PBS/NOG, Ov MBP/10, Ov MBP/11, Ov MBP/29 |
| sero | 427 | IFA test |
| sero | 429 | IHA test |
| eye_general | 29 | Ocular onchocerciasis, no further details |
| eye_general | 131 | History of Eye Disease |
| eye_symptoms | 130 | Eye Discomfort |
| eye_symptoms | 150 | Eye Itching |
| eye_symptoms | 13 | IVIE impaired vision/eye itching |
| eye_symptoms | 141 | Motion Sensitivity Screening Test (MSST) |
| eye_vision-loss | 143 | Visual Impairment |
| eye_vision-loss | 139 | Moderate Visual impairment |
| eye_vision-loss | 140 | Severe Visual impairment |
| eye_vision-loss | 25 | Blindness |
| eye_vision-loss | 411 | Blindness due to oncho |
| eye_vision-loss | 434 | low vision due to oncho |
| eye_vision-loss | 441 | uniocular blindness due to oncho |
| eye_vision-loss | 151 | Uniocular Blindness (only one eye blind) |
| eye_vision-loss | 145 | Night Blindness |
| eye_vision-loss | 146 | Photophobia |
| eye_atrophy/lesions | 300 | oncho related ocular lesions |
| eye_atrophy/lesions | 26 | ocular lesions |
| eye_atrophy/lesions | 301 | severe ocular lesions |
| eye_atrophy/lesions | 15 | Inducible eye lesions |
| eye_atrophy/lesions | 409 | optic atrophy, unspecified |
| eye_atrophy/lesions | 148 | bilateral ocular atrophy |
| eye_atrophy/lesions | 147 | bilateral macular affection |
| eye_atrophy/lesions | 153 | Pterygium |
| eye_atrophy/lesions | 432 | papillary abnormalities |
| eye_visible-mf | 20 | Mf in cornea |
| eye_visible-mf | 133 | Dead microfilarae in cornea (DMFC) |
| eye_visible-mf | 21 | Mf in anterior chamber |
| eye_visible-mf | 24 | Mf in anterior segment |
| eye_visible-mf | 28 | Mf in cornea and/or anterior chamber |
| eye_corneal-disease | 415 | Corneal Opacities |
| eye_corneal-disease | 19 | Punctate Keratitis |
| eye_corneal-disease | 27 | Punctate Keratitis + Mf in anterior chamber |
| eye_corneal-disease | 17 | sclerosing keratitis |
| eye_corneal-disease | 22 | sclerosing keratitis or chorioretinitis in a pattern typical of onchocerciasis |
| eye_corneal-disease | 23 | sclerosing keratitis or chorioretinitis in a pattern typical of onchocerciasis + glaucoma |
| eye_corneal-disease | 149 | corneal scar |
| eye_corneal-disease | 152 | Acute Senilis |
| eye_iris | 16 | Iridocyclitis |
| eye_iris | 403 | pigment epithelial defects |
| eye_iris | 405 | pupillary adhesions |
| eye_iris | 406 | dispersal of pigment in the anterior chamber |
| eye_lens | 135 | Cataract |
| eye_retina | 436 | Intra-retinal pigment changes |
| eye_retina | 437 | Retinal pigment epithelium atrophy |
| eye_retina | 138 | Intra-retinal Disease |
| eye_retina | 142 | Intra-retinal Deposits |
| eye_retina | 438 | white intra-retinal deposits |
| eye_retina | 439 | shiny intra-retinal deposits |
| eye_retina | 410 | Retinal vessel sheathing |
| eye_choroid | 435 | Chorioretinal changes - unspecified |
| eye_choroid | 14 | Onchocercal chorioretinitis |
| eye_choroid | 404 | Chorioretinal atrophy |
| eye_optic-nerve-disease | 144 | Optic nerve atrophy |
| eye_optic-nerve-disease | 18 | Optic nerve disease |
| eye_optic-nerve-disease | 136 | Glaucoma |
| eye_optic-nerve-disease | 407 | Raised intraocular pressure |
| eye_optic-nerve-disease | 137 | Non glaucomatous optic nerve disease |
| skin_general | 423 | Cutaneous Sign |
| skin_general | 40 | Onchodermatitis - general |
| skin_general | 419 | Onchodermatitis - acute |
| skin_general | 420 | Onchodermatitis - chronic |
| skin_general | 52 | Skin Lesions |
| skin_itching | 431 | History of Prolonged Itching |
| skin_itching | 47 | Pruritus |
| skin_itching | 55 | Severe Itching |
| skin_itching | 60 | Scratch Mark |
| skin_rash | 63 | Papular rash |
| skin_rash | 34 | Acute Papular Onchodermatitis |
| skin_rash | 35 | Chronic Papular Onchodermatitis |
| skin_rash | 64 | Pustular rash |
| skin_rash | 65 | Macular Rash |
| skin_rash | 46 | Maculopapular Rashes |
| skin_rash | 400 | papules, unspecified |
| skin_rash | 430 | rash, unspecified |
| skin_lymph-nodes | 53 | Lymphadenopathy |
| skin_lymph-nodes | 424 | Lymphedema |
| skin_lymph-nodes | 442 | Adenolymphocele |
| skin_pigment | 402 | Pigment changes, unspecified |
| skin_pigment | 44 | Leopard Skin |
| skin_pigment | 49 | Depigmentation |
| skin_pigment | 61 | Hypopigmentation |
| skin_pigment | 62 | Hyperpigmentation |
| skin_pigment | 401 | Edematous plaques |
| skin_pigment | 418 | Sowda |
| skin_texture | 41 | Atrophy |
| skin_texture | 66 | Lizard Skin |
| skin_texture | 42 | Scaling |
| skin_texture | 36 | Lichenified Onchodermatitis |
| skin_texture | 433 | hyperkeratosis |
| skin_texture | 440 | Pachydermia |
| skin_inguinal/groin | 70 | Hanging Scrotum |
| skin_inguinal/groin | 71 | Hanging Groin |
| skin_inguinal/groin | 68 | Scrotal Elephantiasis |
| skin_inguinal/groin | 75 | Hydroceles |
| skin_inguinal/groin | 74 | Hypertrophy of the scrotal skin |
| skin_inguinal/groin | 67 | Hernia |
| skin_legs | 58 | Elephantiasis |
| skin_legs | 413 | severe unilateral leg involvement |
| other | 422 | DEC Patch Test |
| other | 59 | Rheumatism |
| other | 91 | Knott's method |
| other | 93 | LAMP (loop-mediated isothermal amplification) (Turbidity) |
| other | 94 | LAMP (loop-mediated isothermal amplification) (Color) |
| other | 425 | Blood smear, thick |
| other | 428 | Urine test |

Caption: A table showing the definition of diagnostic codes used in the final dataset to diagnostic type and name.
